# Are teachers' knowledge of dyslexia, perceived teaching ability and warmth during teaching related to Chinese students' reading development?

**DOI:** 10.1111/bjep.70103

**Published:** 2026-06-23

**Authors:** Anna Jia‐Jun Zhang, Tomohiro Inoue

**Affiliations:** ^1^ Department of Psychology The Chinese University of Hong Kong Hong Kong China; ^2^ Centre for Developmental Psychology The Chinese University of Hong Kong Hong Kong China

**Keywords:** Chinese, perceived ability to teach reading, reading development, teachers' knowledge of dyslexia, warmth during teaching

## Abstract

**Background:**

Teachers' characteristics have been linked to their students' reading development. However, previous research has primarily focused on the early phase of reading development in Western societies.

**Aims:**

We examined the associations between teachers' knowledge of dyslexia, perceived ability to teach reading and warmth during teaching and Chinese students' reading development across primary grades.

**Sample:**

Fifty‐four Chinese language arts teachers and their 1517 students in Grades 1 to 5 (mean age = 8.63 years, *SD* = 1.39, 51.2% female) participated in the study.

**Method:**

The teachers completed a questionnaire assessing their knowledge of dyslexia, perceived ability to teach reading and warmth during teaching. The students were assessed on their reading skills (sentence reading efficiency and passage reading comprehension) in the middle and at the end of the school year.

**Results:**

Multilevel modelling showed that teachers' perceived ability to teach reading was associated with their students' reading gains over time after controlling for other teacher variables and students' age, gender and earlier reading skills. In contrast, their knowledge of dyslexia was not significantly associated with students' reading skills, either for the whole class or for lower‐performing students.

**Conclusion:**

These findings suggest that teachers' self‐efficacy (operationalized as perceived teaching ability) may play a role in Chinese students' reading development.

## INTRODUCTION

Learning to read requires explicit formal instruction, and teachers are undoubtedly the primary agents in this endeavour (Aaron et al., 2008). A growing body of research has suggested that teachers' characteristics, including their knowledge about various language and literacy concepts and perceived ability to teach reading, are associated with their students' reading development (e.g., McCutchen et al., 2009; Parrila et al., 2024; Podhajski et al., 2009). Beyond these attributes, some researchers have suggested that teachers' warmth and supportive teaching styles can enhance students' learning by influencing their motivation and engagement (e.g., de Wilde et al., 2016; Rickert & Skinner, 2022; Wentzel, 2015).

However, most previous studies examining the role of teacher characteristics in students' reading development have been conducted in Western societies, leaving it unknown whether the teacher characteristics can similarly predict students' reading outcomes in non‐Western contexts, such as China. Furthermore, most studies examining the role of teacher characteristics have focused on reading outcomes at the word level during the early years of primary school (e.g., Parrila et al., 2024; Piasta et al., 2009). Although this is important and reasonable both practically and methodologically, this limits our understanding of the effects of teacher characteristics on students' reading skills at the sentence and passage levels beyond these years. To address these gaps, we examined the roles of teachers' knowledge of reading difficulties (i.e., dyslexia), perceived ability to teach reading and warmth during teaching in predicting their students' reading gains over time in a longitudinal sample of Chinese students in Grades 1–5.

In the present study, we conceptualize these teacher characteristics as complementary dimensions that may support students' reading development through different, but potentially overlapping, pathways (see Figure 1 for a conceptual model). Teachers' knowledge of dyslexia represents a cognitive/professional knowledge dimension that informs how teachers recognize reading difficulties and respond with instruction (e.g., Parrila et al., 2024). In turn, teachers' perceived ability to teach reading represents a motivational/self‐efficacy dimension that shapes their instructional effort, persistence and willingness to adapt their teaching to students' learning needs (e.g., Guo et al., 2012). Finally, teachers' warmth during teaching represents a relational‐emotional dimension that can foster students' engagement, sense of security and motivation to participate in learning (e.g., Bergin & Bergin, 2009). Importantly, these teacher characteristics are expected to contribute to students' achievement through partially different pathways, including teachers' instructional practices and efforts, as well as students' motivation and engagement.

**FIGURE 1:**
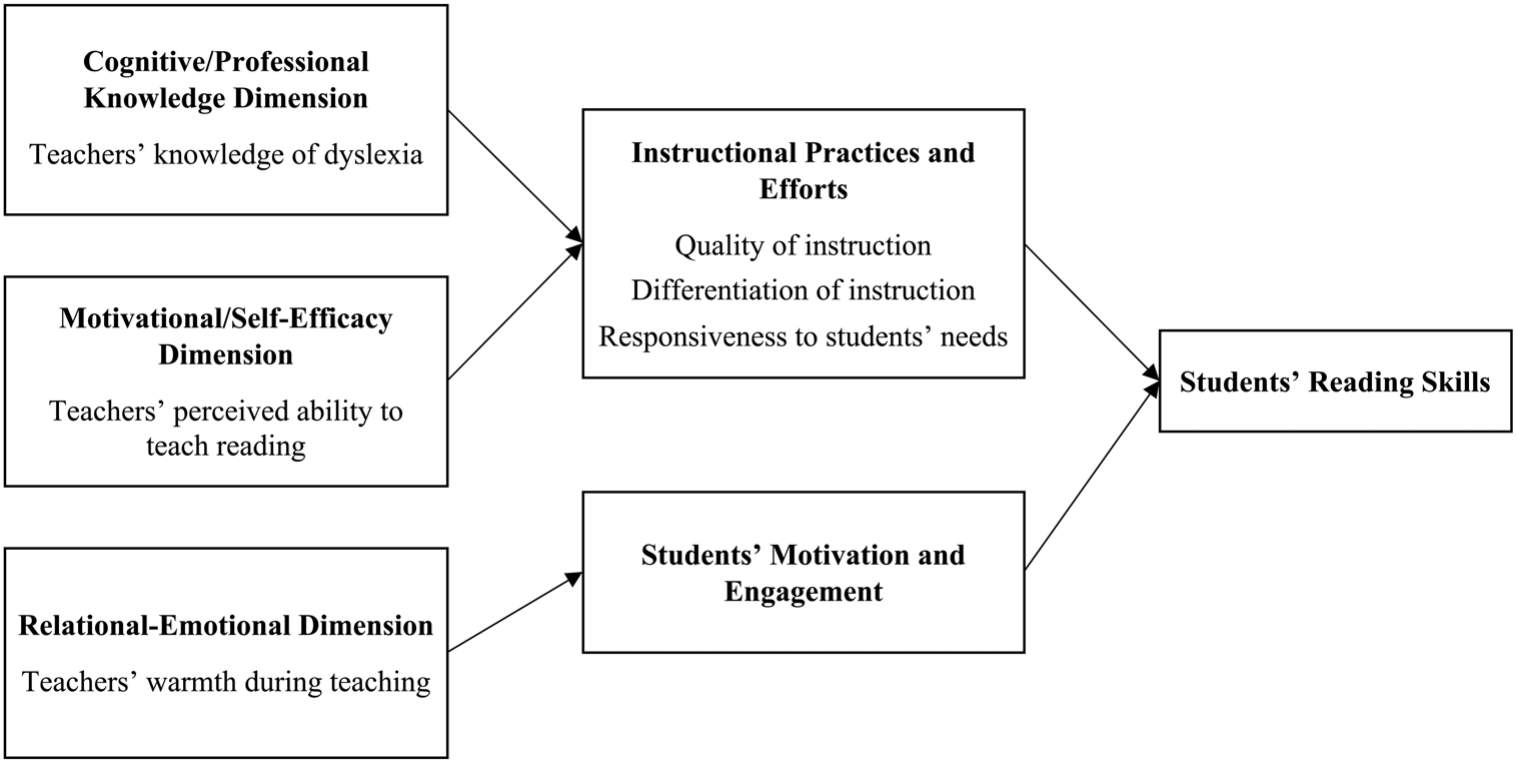
The conceptual model for the associations between teachers' knowledge of dyslexia, perceived ability to teach reading, warmth during reaching and students' reading skills.

Empirically testing the predictive roles of these teacher characteristics in students' reading development is theoretically important for two reasons. First, building upon Shulman's (1986) model of teacher knowledge and Desimone's (2009) framework of teacher professional development, we can examine whether teachers' knowledge and abilities in fostering students' reading development are applicable in the Chinese context. In fact, to our best knowledge, this study is among the first to examine the effects of teachers' characteristics on students' reading in a Chinese context. Second, it allows us to examine whether the effects of teachers' warmth during teaching on students' learning generalize to their reading development (see Howard et al., 2024, for a recent meta‐analysis). Motivation theories in learning (e.g., self‐determination theory; Ryan & Deci, 2000) have conceptualized that warm and supportive classroom climates can foster students' engagement and learning. However, there is still a paucity of studies that have directly examined this effect in the reading domain (see Hu et al., 2018; Ly et al., 2012; Pakarinen et al., 2017, for exceptions).

### Dyslexia in China and teachers' knowledge of dyslexia

While dyslexia has been observed across languages and writing systems (Catts et al., 2026; Verhoeven et al., 2019; Yang et al., 2022), research and practice on dyslexia in China lags far behind that of English‐speaking countries, where research has been ongoing for over a century (Lin et al., 2020). In fact, public awareness of dyslexia and official efforts to support individuals with special education needs in China have only begun in the past decade (Ministry of Education of the People's Republic of China, 2017). This significant lag in research, practice and policy development is particularly concerning given that Chinese is one of the most widely spoken languages in the world, making the study of reading difficulties and dyslexia in this linguistic context both urgent and globally relevant (Martin et al., 2023; McBride, 2016).

Chinese is a morphosyllabic writing system in which reading relies heavily on orthographic and morphological processing, as well as on phonological processing (see Ruan et al., 2018; Yang et al., 2013, for meta‐analyses). Because of these characteristics, reading development in Chinese is highly cognitively demanding and extends over a longer developmental period, and Chinese dyslexia may therefore manifest differently than in alphabetic orthographies (e.g., McBride, 2016; McBride et al., 2018). In fact, as Chinese children must learn thousands of visually complex characters throughout their school years, the primary difficulties of dyslexia often manifest as challenges in acquiring accurate word recognition skills.

While the diagnosis of dyslexia falls outside a teacher's responsibilities, it is important that they have a working knowledge of dyslexia. This is because teachers' early identification, explicit instruction and timely targeted intervention are key factors that can significantly enhance the academic success of children with dyslexia, despite the lifelong nature of the disorder (Knight, 2018; Moats, 2009; Washburn et al., 2014). However, previous studies in Western societies have shown that teachers may have both correct and incorrect understandings of dyslexia (e.g., Soriano‐Ferrer et al., 2016; Wadlington & Wadlington, 2005). For example, although teachers often correctly understand that individuals with dyslexia have language‐based difficulties, such as decoding difficulties, they tend to misunderstand that seeing letters and words backwards is a characteristic of dyslexia. Similar results have been reported among Chinese teachers, showing relatively limited knowledge of dyslexia and common misconceptions (Yin et al., 2019).

Additionally, teachers' knowledge of dyslexia may be relevant not only to students with reading difficulties but also to all learners in their classrooms, possibly indirectly through their classroom instruction. Specifically, teachers who understand that dyslexia is primarily characterized by word‐level reading difficulties are more likely to be aware of the importance of explicit and systematic instruction of foundational literacy skills, such as decoding (Hudson et al., 2021; Moats, 2009; Piasta et al., 2009; see Georgiou et al., 2026, for a meta‐analysis). This may improve the quality of their classroom instruction, known as Tier 1 instruction in the frameworks of response to intervention (RTI; Fuchs & Fuchs, 2006) and Multi‐Tiered Systems of Support (MTSS; McIntosh & Goodman, 2016), which, in turn, benefits all of their students. Several studies have shown that teachers' professional knowledge, including their knowledge of dyslexia, influences their classroom instructional practices, including the quality and differentiation of instruction (effectively modifying the instruction according to the students' needs), progress monitoring and responsiveness to students' emerging difficulties (McCutchen et al., 2009; Parrila et al., 2024; Piasta et al., 2009). Based on this existing evidence, one might expect that teachers' knowledge of dyslexia would indirectly predict the average reading growth of their students through their instructional practices (see Figure 1).

However, very few studies, to our knowledge, have examined the impact of teachers' knowledge of dyslexia on their students' reading outcomes. To date, only one study (Parrila et al., 2024) has included questions on teachers' knowledge of dyslexia, but these were embedded within a broader measure of teachers' knowledge of various language and literacy concepts (e.g., phonological awareness, phonics; see Appendix 1 in Parrila et al., 2024, p. 1177, for their teacher knowledge measure). While they reported a positive association between teachers' knowledge of language and literacy concepts and students' reading skills, it remains unclear whether this relationship holds when examining their knowledge of dyslexia independently. It is therefore important to examine teachers' knowledge of dyslexia separately, as this can help clarify whether this specific domain of professional knowledge contributes to students' reading development in its own right. This distinction may also provide insights for designing more optimized teacher training and professional development programmes (Moats, 2009; Piasta et al., 2009; Yin et al., 2019).

### Perceived ability to teach reading and warmth during teaching

Another line of research that has gained researchers' attention is the effect of teachers' self‐efficacy, often operationalized with their perceived ability to teach reading, on their students' reading outcomes (e.g., Aro & Björn, 2016; Caprara et al., 2006; Guilbault et al., 2025, 2026; Parrila et al., 2024). Drawing on Bandura's (1986) social cognitive theory, teachers' self‐efficacy is defined as their belief in their ability to perform teaching tasks successfully. A high level of self‐efficacy is often considered an important characteristic of effective teachers, because it motivates them to make greater instructional efforts to facilitate their students' learning (e.g., Tschannen‐Moran & Johnson, 2011).

However, previous studies have provided mixed findings regarding the association between teachers' self‐efficacy and students' reading development (e.g., Aro & Björn, 2016; Cantrell et al., 2013; Guo et al., 2012; Prewett & Whitney, 2021). For example, Guo et al. (2012) showed that American teachers' self‐efficacy indirectly predicted fifth graders' reading skills through teachers' instructional support. Similarly, Caprara et al. (2006) found that Italian teachers' self‐efficacy predicted students' reading achievement over time, even after controlling for students' initial reading ability. In contrast, Prewett and Whitney (2021) reported no significant association between American teachers' self‐efficacy and their eighth graders' reading achievement. Cantrell et al. (2013) also found that teachers' self‐efficacy was not significantly associated with their students' reading or vocabulary skills.

In addition to the effects of teachers' knowledge and self‐efficacy, some theories suggest that their warmth during teaching may also facilitate students' learning outcomes, including reading skills, possibly by influencing their motivation and engagement (e.g., Bergin & Bergin, 2009; Pianta, 1999; Ryan & Deci, 2000). Extended attachment theory posits that when students experience emotional security in their relationships with teachers, they are more likely to engage in exploratory learning and inquiry without fear, thereby promoting learning (Bergin & Bergin, 2009; Pianta, 1999). Self‐determination theory (Ryan & Deci, 2000) provides an alternative mechanism, suggesting that when teachers are sensitive to students' learning needs and provide emotional support, they fulfil students' basic psychological need for relatedness, which, in turn, can foster students' motivation and engagement in learning (Hamre et al., 2013). It is important to note that these theoretical accounts imply an indirect pathway: Teachers' warmth during teaching is assumed to foster students' learning primarily by increasing behavioural, emotional and cognitive engagement, thereby improving learning outcomes.

Several empirical studies have supported these predictions (e.g., Gregory & Korth, 2016; Sakiz et al., 2012; see Quin, 2017, and Roorda et al., 2011, for meta‐analyses). For example, in a longitudinal study with 15,362 high school students, Fall and Roberts (2012) showed that students' perceived care and support from their teachers predicted students' self‐perceptions of control over outcomes in schools and interest in and satisfaction with school, which in turn predicted their academic engagement and achievement (a latent variable included reading and math). To date, only a few studies have directly examined this link in students' reading development (Hu et al., 2018; Ly et al., 2012; Pakarinen et al., 2017). Pakarinen et al. (2017) found that Finnish first graders whose teachers provided greater emotional support showed better reading skills at the end of Grade 1 (see Ly et al., 2012, for similar findings). Taken together, these findings suggest that teachers' warmth during teaching can play a role in indirectly shaping their students' learning in various domains, including reading. Given the central role of teachers in literacy learning in classrooms, it is essential to investigate whether these associations also extend to non‐Western settings, such as Chinese contexts.

Importantly, Chinese classrooms differ from their Western counterparts due to the enduring influence of Confucian heritage culture, which emphasizes respect for authority, academic diligence and collective success (Li, 2003; Tweed & Lehman, 2002). Within this cultural framework, teachers are regarded not only as academic instructors but also as moral exemplars and role models responsible for students' holistic development (Li, 2012). In these hierarchical teacher–student relationships, where students are expected to follow rather than question authority (Xu et al., 2023), the role of teachers' perceived ability to teach reading may be relatively more prominent than in other cultural contexts, whereas the role of teachers' warmth may be moderate. Despite this possibility, empirical evidence on the roles of teachers' perceived ability to teach reading and warmth during teaching in Chinese students' reading development remains scarce. To our knowledge, only one study has directly examined this association and found that teachers' emotional support positively contributed to Chinese kindergarteners' reading and vocabulary learning (Hu et al., 2018). More research is therefore needed to investigate whether and how teachers' perceived ability and warmth can contribute to students' reading development in Chinese contexts. Investigating their associations in Chinese not only fills the gap in the literature but also informs whether the theoretical links between these teacher characteristics and students' reading development generalize across linguistically and culturally distinct settings.

### The present study

The purpose of this longitudinal study was to examine the roles of Chinese teachers' knowledge of dyslexia, perceived ability to teach reading and warmth during teaching in predicting their students' reading development in Chinese. We conceptualized these teacher characteristics as representing cognitive/professional knowledge, motivational/self‐efficacy and relational‐emotional dimensions, respectively (see Figure 1). To capture the effects of teacher characteristics on students' reading skills beyond the word level across the primary years, we assessed Grade 1–5 students' reading skills in sentence reading efficiency and passage reading comprehension. In addition, we examined the effects of teacher characteristics on students' reading skills at both the whole‐class level and among students with different performance levels (good, average and poor readers) using two sets of multilevel modelling approaches. Importantly, when examining the effects of the teacher variables, we statistically controlled for students' age, gender, earlier reading skill (autoregressor) and teachers' years of teaching experience. By doing so, we sought to examine whether and how teachers' knowledge of dyslexia, perceived teaching ability and warmth during teaching contribute to students' reading gains over time, above and beyond what can be explained by the other variables, including students' earlier skill levels.

Based on the findings from previous studies reviewed above (e.g., Caprara et al., 2006; Fall & Roberts, 2012; Guo et al., 2012; Parrila et al., 2024; Rickert & Skinner, 2022), we hypothesized that teachers' perceived ability to teach reading and warmth during teaching would predict students' later reading skills over and above the effects of the other teacher and student variables. Additionally, although no previous studies in Chinese have examined the effect of teachers' knowledge of dyslexia on their students' reading development, we expected, based on the findings from studies in other languages (e.g., Parrila et al., 2024; Washburn et al., 2011), that there would be a positive association between the two at the whole‐class level, and the association would be particularly evident for lower‐performing students.

## METHOD

### Participants

The present study is a pre‐registered data analysis from an ongoing project designed to examine the developmental trajectories of Chinese children's literacy and numeracy skills throughout the primary school years. All analyses were registered at the Open Science Framework (OSF), and the analysis code, including our model specifications and final model results, has been made publicly available at https://osf.io/vuxh4/. We do not have permission to share data at the level of individual participants. The study was conducted in accordance with the ethical standards of the American Psychological Association (American Psychological Association, 2017). Ethics approval for this project has been obtained from the Survey and Behavioural Research Ethics Committee of The Chinese University of Hong Kong (Reference No. SBRE‐24‐0008).

Our sample for this study consisted of 54 Chinese language arts teachers from three public schools in Wenzhou, China, and their 1517 students (741 boys and 776 girls; mean age = 8.63 years, *SD* = 1.39, range = 6.25–12.25) from 54 classes in Grades 1–5. Each classroom corresponded to a teacher, so students were nested within the classrooms/teachers. The three participating schools from which we recruited our participants were located in three districts of Wenzhou. These are all public schools that follow the national curriculum standards established by the Ministry of Education (Ministry of Education of the People's Republic of China, 2022) and reflect typical school settings in China, with structured literacy instruction and consistent teaching practices across classrooms. The teachers were mostly female (87.0%), with most teachers holding a bachelor's degree (83.3%) and a smaller proportion having attained a master's degree (11.1%) or a high school diploma (5.6%). The students were tested on their reading skills in the middle (Time 1) and at the end of the school year (Time 2). All students were native speakers of Mandarin Chinese and were reported by their schools to have no documented learning, emotional or sensory difficulties. Prior to data collection, consent was obtained from students, parents, teachers and schools.

Literacy education in China is designated as a core subject throughout compulsory education and is shaped by a centralized education system that places strong emphasis on early and systematic reading instruction. National curriculum standards specify clear grade‐level expectations, including character recognition, vocabulary development, oral reading fluency and the progressive development of reading comprehension skills (Ministry of Education of the People's Republic of China, 2022). For example, students in Grades 1–2 are expected to recognize 1600 characters, whereas those in Grades 3–4 are expected to recognize 2500 characters. In addition, teaching practices are standardized across schools, supported by nationally prescribed curriculum materials and textbooks, which contribute to a consistent instructional environment.

### Measures

#### Students' measures

##### Reading skills

Students' reading skills were assessed in terms of two aspects: sentence reading efficiency and passage reading comprehension. *Sentence reading efficiency* was assessed using a sentence verification task adapted from Lei et al. (2011). This task has been used in several Chinese studies with students across grade levels (e.g., Pan et al., 2016; Xue et al., 2013; Zhang et al., 2024), showing good reliability and validity evidence. Students were given one minute to silently read as many of the 60 short sentences (e.g., A fish lives on the land) as possible and judge the truthfulness of each sentence by circling ‘Y’ (for Yes) or ‘N’ (for No). A practice trial consisting of six items was conducted prior to the formal testing to ensure familiarity. A participant's score was calculated by the number of correct items minus the number of incorrect items (max = 60). The correlation between the two time points was .63, indicating high stability of the measure.

*Passage reading comprehension* was assessed using a reading comprehension task adapted from Ho et al. (2017). This task has been used in several Chinese studies with students across grade levels (e.g., Ho et al., 2018; Lui et al., 2021), showing good reliability and validity evidence. The task consisted of four passages, each followed by four or five multiple‐choice questions and one to four open‐ended questions (a total of 17 multiple‐choice questions and 12 open‐ended questions). Due to the limited writing abilities among young children, students in Grades 1 and 2 were required to answer only multiple‐choice questions, while those in Grades 3–5 were required to complete both multiple‐choice and open‐ended questions. This decision was made based on discussions with their Chinese language arts teachers to ensure that the assessment was age‐appropriate and minimized the burden for younger children. For the multiple‐choice questions, one point was given for a correct response (max = 17). For the open‐ended questions, children wrote down their responses, and these were scored according to the guidelines of Ho et al. (2017) by a trained research assistant (max = 15). To ensure scoring reliability, a second trained rater independently scored a subset of responses (8.5% of the sample), yielding high inter‐rater consistency (*r* = .95 and .96 for Time 1 and Time 2, respectively). Any discrepancies or ambiguous responses were discussed between the raters until consensus was reached, following the predefined scoring criteria. The raw scores for each part (i.e., multiple‐choice and open‐ended questions) were converted to *z*‐scores separately at each time point using the whole sample. For children in Grades 1 and 2, a participant's score was their *z*‐score for the multiple‐choice questions. For children in Grades 3–5, the score was calculated by averaging the *z*‐scores for both multiple‐choice and open‐ended questions. Cronbach's alpha reliability in our sample was .61 to .70 for Grades 1 and 2 and .83 to .84 for Grades 3–5.

Following our pre‐registered analysis plan, we calculated a composite score for students' reading skills by averaging the *z*‐scores of the two reading measures and used it in the analyses to reduce the potential effect of task‐specific variability. The two reading measures were strongly correlated across time points (*r* = .61 to .68), suggesting the convergent validity of our reading measures for students' sentence and passage reading skills.

#### Teachers' measures

##### Knowledge of dyslexia

Teachers' knowledge of dyslexia was assessed using questions adapted from Parrila et al. (2024). Teachers were asked to select the response they believed best fit each statement. Response options included the following: 1 = *definitely false*, 2 = *possibly false*, 3 = *possibly true* and 4 = *definitely true*. The four items included: (a) seeing letters and words backwards is a characteristic of dyslexia; (b) children with dyslexia can be helped by using coloured lenses/overlays; (c) children with dyslexia have problems in decoding and spelling but not in listening comprehension; (d) dyslexics tend to have lower IQ scores than non‐dyslexics. Each item was scored such that two points were awarded for a fully correct response (i.e., the precise correct answer), and one point was awarded for a partially correct response that was directionally accurate (e.g., selecting ‘possibly false’ when the correct answer was ‘definitely false’). A composite score was calculated by summing the points given from the four items (max = 8). Cronbach's alpha reliability in our sample was .62.

##### Perceived ability to teach Reading

Teachers' perceived ability to teach reading was assessed using questions adapted from Washburn et al. (2011) and Parrila et al. (2024). The teachers were asked to rate themselves on a 4‐point Likert scale, ranging from 1 = *minimal* to 4 = *expert*, to indicate their perceptions of their own teaching abilities. The items included teaching (a) reading to typically developing children, (b) reading to struggling readers, (c) radical‐related knowledge and (d) vocabulary‐related knowledge. A composite score was calculated by summing the points of the four questions (max = 16). Cronbach's alpha reliability in our sample was .81.

##### Warmth during teaching

Teachers' warmth during teaching was assessed using questions adapted from Kuntsche et al. (2006). The teachers were asked to rate themselves on a 5‐point Likert scale, ranging from 0 = *strongly disagree* to 4 = *strongly agree*, to indicate the levels of warmth and support in their teaching. The items included: (a) I comfort students when they are having difficulties; (b) I encourage students when they score badly; (c) I have a good relationship with students; (d) I am there for students if they need help; (e) I congratulate students when they have done something well; (f) I value students' opinions even if they differ from mine; and (g) I explain why I have asked students to do something. A composite score was calculated by summing the points of the seven questions (max = 28). Cronbach's alpha reliability in our sample was .80.

##### Years of teaching experience

Years of teaching experience were obtained by asking teachers to report their years of teaching in numbers.

### Procedure

The students were assessed on their reading skills with a six‐month interval in the middle (December 2024; Time 1) and at the end of the school year (June 2025; Time 2). Testing was administered on a group basis by trained research assistants and completed in a single session lasting approximately 50 min, with a 10‐min break between the two tasks. The teachers responded to the questionnaires on their knowledge of dyslexia, perceived ability to teach reading, warmth during teaching and years of teaching experience at Time 1, around the same time as their students' testing.

### Statistical analysis

To examine the roles of teacher variables in predicting their students' reading gains over time, we employed a multilevel modelling approach (Heck & Thomas, 2009). This allowed us to differentiate variances in the variables into two distinct levels: The first level describes variance due to differences between teachers (i.e., between‐classroom variation); the second level describes variance due to individual differences between students (i.e., within‐classroom variation). All analyses were conducted using the *lmer* function in the *lmerTes*t package in R (Kuznetsova et al., 2017; R Core Team, 2025), and teacher variables were grand‐mean centred prior to model estimation.

For the between‐classroom predictors, the model included teachers' knowledge of dyslexia, perceived ability to teach reading and warmth during teaching, along with teachers' years of teaching experience as a covariate. For the within‐classroom predictors, the model included students' age, gender and reading skills at Time 1 (i.e., autoregressor). Students' reading skills at Time 2 were treated as the outcome variable at both levels, and a random intercept was included to capture unobserved between‐classroom variances. All predictors were entered as fixed effects to evaluate their unique contributions to students' reading skills at Time 2.

## RESULTS

### Descriptive statistics

Table 1 shows the descriptive statistics for all measures used in the study. Overall, the teachers in our sample reported a slightly higher perceived ability to teach reading compared to what has been reported in previous studies of teachers in North America (Cunningham et al., 2004; Parrila et al., 2024; Washburn et al., 2011) and New Zealand (Arrow et al., 2019). On the other hand, the proportion of the maximum possible score of .53 in their knowledge of dyslexia was comparable to .52 reported by Washburn et al. (2011) for American teachers. This suggests that Chinese teachers, like their Western counterparts, possess some accurate knowledge about dyslexia, but also hold misconceptions (see Yin et al., 2019, for similar results).

**TABLE 1:** Descriptive statistics for the measures used in the study.

Variables	*n*	Mean	*SD*	Range	Skew	Kurtosis	ICC
Student variables							
Age in months_T1	1517	103.59	16.62	75–147	−1.11	0.43	–
Sentence reading efficiency_T1 (60)	1517	18.44	11.31	0–53	0.44	0.05	.53 [.44, .64]
Sentence reading efficiency_T2 (60)	1517	22.79	11.37	0–57	0.41	0.06	.47 [.38, .58]
Passage reading comprehension_MC_T1 (17)	1517	7.12	3.96	0–17	0.27	−0.76	.40 [.32, .51]
Passage reading comprehension_MC_T2 (17)	1517	8.03	3.98	0–17	0.10	−0.93	.36 [.28, .47]
Passage reading comprehension_OE_T1 (15)	788	6.67	3.80	0–14.5	−0.09	−0.88	.27 [.19, .41]
Passage reading comprehension_OE_T2 (15)	788	7.04	3.36	0–13.5	−0.26	−0.51	.20 [.13, .32]
Teacher variables							
Knowledge of dyslexia_T1 (8)	54	4.24	1.71	1–7	−0.06	−1.10	–
Perceived ability to teach reading_T1* (16)	54	10.80	1.97	6–15	−0.26	−0.55	–
Warmth during teaching_T1** (28)	54	26.24	2.40	17–28	−1.67	2.59	–
Years of teaching experience_T1	54	18.59	10.3	0–46	0.10	−0.43	–

*Note*: Numerals in parentheses represent the possible maximum score for each variable.

Abbreviations: ICC, intraclass correlation; MC, multiple‐choice; OE, open‐ended; T1, Time 1; T2, Time 2.

* Perceived ability to teach reading has four options, ranging from 1 = *minimal* to 4 = *expert*.

** Warmth during teaching has five options, ranging from 0 = *strongly disagree* to 4 = *strongly agree*.

Tables 2 and 3 show the correlations among the variables at the teacher (classroom) and student levels, respectively. Teacher‐level correlations were calculated using class‐average scores, while student‐level correlations were calculated using individual students' scores. Students' reading skills were strongly correlated with each other and across two time points at both levels (*r* = .66 to .94 at the teacher level and .53 to .88 at the student level), indicating that both students' and classrooms' relative performance levels were fairly stable over time. Teachers' perceived ability to teach reading was correlated with both aspects of students' reading skills at Time 2 (*r* = .32 for sentence reading efficiency and .23 for multiple‐choice questions in passage reading comprehension). Teachers' years of teaching experience were also correlated with students' passage reading comprehension at Time 2 (*r* = .25 to .31). In contrast, teachers' knowledge of dyslexia was not correlated with their students' average reading scores (*r* = −.06 to .05).

**TABLE 2:** Teacher‐level correlations among the observed variables.

Variables	1.	2.	3.	4.	5.	6.	7.	8.	9.	10.
***Student variables*** ^a^										
1. Age_T1										
2. Sentence reading efficiency_T1	80*** [68, 88]									
3. Sentence reading efficiency_T2	71*** [55, 82]	76*** [62, 85]								
4. Passage reading comprehension_MC_T1	93*** [89, .96]	79*** [66, 87]	77*** [63, 86]							
5. Passage reading comprehension_MC_T2	80*** [67, 88]	74*** [60, 84]	78*** [64, 87]	85*** [76, 91]						
6. Passage reading comprehension_OE_T1	93*** [88, 96]	79*** [66, 87]	66*** [48, 79]	88*** [81, 93]	75*** [60, 85]					
7. Passage reading comprehension_OE_T2	88*** [80, 93]	76*** [61, 85]	66*** [48, .79]	83*** [72, 90]	83*** [72, 90]	94*** [90, 96]				
***Teacher variables***										
8. Knowledge of dyslexia_T1	−02 [−28, 25]	02 [−25, 29]	−06 [−32, 21]	01 [−26, 28]	01 [−26, 28]	05 [−22, 31]	02 [−25, 29]			
9. Perceived ability to teach reading_T1	07 [−20, 33]	23* [−04, 47]	32* [06, 54]	08 [−19, 34]	.23*[02, 48]	08 [−20, 34]	13 [−14, 39]	−04 [−31, 23]		
10. Warmth during teaching_T1	08 [−19, 34]	10 [−17, 36]	12 [−16, 37]	05 [−22, 31]	02 [−25, 28]	08 [−20, 34]	05 [−22, 31]	20 [−07, 44]	21 [−06, 46]	
11. Years of teaching experience_T1	16 [−12, 41]	03 [−24, 29]	18 [−09, 43]	16 [−11, 41]	31* [04, 53]	13 [−14, 38]	25* [01, 49]	−13 [−39, 14]	21 [−06, 45]	29* [02, 52]

Abbreviations: MC, multiple‐choice; OE, open‐ended; T1, Time 1; T2, Time 2.

^a^ Correlations were calculated using classroom average scores (*N* = 54).

* *p* < .05.

** *p* < .01.

*** *p* < .001.

**TABLE 3:** Student‐level correlations among the observed variables.

Variables	1.	2.	3.	4.	5.	6.
1. Age_T1						
2. Sentence reading efficiency_T1	61*** [57, 64]					
3. Sentence reading efficiency_T2	50*** [46, 53]	63*** [60, 66]				
4. Passage reading comprehension_MC_T1	61*** [58, 64]	59*** [56, 62]	58*** [55, 62]			
5. Passage reading comprehension_MC_T2	54*** [50, 57]	58*** [55, 61]	62*** [58, 65]	69*** [66, 71]		
6. Passage reading comprehension_OE_T1	79*** [77, 81]	64*** [61, 67]	53*** [49, 56]	68*** [65, 71]	58*** [54, 61]	
7. Passage reading comprehension_OE_T2	79*** [77, 80]	62** [59, 65]	54*** [51, 58]	63*** [60, 66]	61*** [58, 65]	88*** [86, 89]

*Note*: Correlations were calculated using individual students' scores (*N* = 1517).

Abbreviations: MC, multiple‐choice; OE, open‐ended; T1, Time 1; T2, Time 2.

** *p* < .01.

*** *p* < .001.

### Multilevel modelling

Table 4 shows a summary of the multilevel model predicting students' later reading skills by teacher‐ and student‐level predictors at Time 1. The model explained a substantial proportion of variance in students' reading skills at Time 2 (marginal *R* ^2^ = .69; conditional *R* ^2^ = .79). It should be noted, however, that much of this explained variance is likely attributable to students' reading skills at Time 1 (i.e., autoregressor). At the student level, students' gender (β = −.04, 95% CI [−0.06, −0.01], *p* = .002) and reading skills at Time 1 (β = .76, 95% CI [.72, .79], *p* < .001) predicted their reading skills at Time 2. The negative coefficient for gender indicated that, in our sample, girls slightly outperformed boys. At the teacher level, teachers' perceived ability to teach reading independently predicted their students' reading skills at Time 2 (β = .10, 95% CI [.01, .20], *p* = .047) after controlling for all other teacher and student variables in the model. In addition, teachers' years of teaching experience predicted students' reading skills at Time 2 (β = .13, 95% CI [.03, .23], *p* = .018). In contrast, teachers' knowledge of dyslexia and warmth during teaching were not significantly associated with students' reading skills at Time 2 when the effects of the other variables were controlled.

**TABLE 4:** Hierarchical linear modelling for students' reading skills at Time 2.

Fixed effects	Stand. estimate	*SE*	BC bootstrapped 95% CI	*p*
Student level				
Intercept	0.02	0.05	[−0.07, 0.12]	.148
Age_T1	0.07	0.04	[−0.01, 0.14]	.080
Gender_T1^a^	−0.04	0.01	[−0.06, −0.01]	.002
Reading skills_T1	0.76	0.02	[0.72, 0.79]	< .001
Teacher level				
Knowledge of dyslexia_T1	0.00	0.05	[−0.09, 0.09]	.957
Perceived ability to teach reading_T1	0.10	0.05	[0.01, 0.20]	.047
Warmth during teaching_T1	−0.04	0.05	[−0.14, 0.05]	.388
Years of teaching experience_T1	0.13	0.05	[0.03, 0.23]	.018
Random effects	Variance	*SD*		
Intercept	0.08	0.28		
Residual	0.16	0.40		
Model metrics	Value			
Marginal *R* ^2^	0.69			
Conditional *R* ^2^	0.79			

Abbreviation: BC, bias‐corrected; T1, Time 1.

^a^ Students' gender was coded as 1 for male and 0 for female.

### Post‐hoc robustness checks

Next, we fitted a series of multilevel quantile regression models to test whether the effects of teachers' knowledge of dyslexia vary across students with different reading skill levels (i.e., 25th, 50th and 75th percentiles of the reading skill distribution; Koenker & Hallock, 2001). For clarity, we note that this analysis was not originally included in our pre‐registered analysis plan and should therefore be viewed as exploratory. We placed particular focus on lower‐performing children (i.e., those in the bottom 25th percentile) because we expected that the effect of teachers' knowledge of dyslexia on students' outcomes would be particularly evident for this group. Given the model's complexity, we removed students' gender and teachers' warmth during teaching due to their limited relevance to the aim of this analysis. The results showed that neither teachers' perceived ability to teach reading nor their knowledge of dyslexia was significantly associated with students' reading skills at either quantile, including lower‐performing readers (see the results for τ = 0.25 in Table 5). These results were replicated when we ran the same models with students' gender and teachers' warmth during teaching as additional covariates.

**TABLE 5:** Hierarchical quantile regression modelling for students' reading skills at Time 2.

Parameter	Standard estimate	*SE*	BC bootstrapped 95% CI	*p*
**τ = 0.25**				
Student level				
Intercept	−0.36	0.31	[−0.96, 0.24]	.312
Age_T1	0.00	0.00	[0.00, 0.01]	.780
Reading skills_T1	0.80	0.05	[0.70, 0.89]	< .001
Teacher level				
Knowledge of dyslexia_T1	0.02	0.03	[−0.04, 0.08]	.614
Perceived ability to teach reading_T1	0.01	0.03	[−0.05, 0.08]	.716
Years of teaching experience_T1	0.00	0.01	[−0.01, 0.02]	.704
**τ = 0.50**				
Student level				
Intercept	−0.23	0.29	[−0.80, 0.34]	.473
Age_T1	0.00	0.00	[0.00, 0.01]	.488
Reading skills_T1	0.84	0.05	[0.75, 0.93]	< .001
Teacher level				
Knowledge of dyslexia_T1	0.03	0.03	[−0.03 0.09]	.307
Perceived ability to teach reading_T1	0.02	0.02	[−0.02, 0.06]	.392
Years of teaching experience_T1	0.00	0.00	[0.00, 0.01]	.272
**τ = 0.75**				
Student level				
Intercept	0.23	0.36	[−0.48, 0.93]	.511
Age_T1	0.00	0.01	[−0.01, 0.01]	.969
Reading skills_T1	0.78	0.04	[0.70, 0.86]	< .001
Teacher level				
Knowledge of dyslexia_T1	0.05	0.03	[−0.01, 0.11]	.156
Perceived ability to teach reading_T1	0.02	0.02	[−0.02, 0.06]	.316
Years of teaching experience_T1	0.01	0.01	[0.00, 0.02]	.168

Abbreviation: BC, bias‐corrected; T1, Time 1.

Additionally, to explore possible associations between teachers' variables and students' reading outcomes (i.e., sentence reading proficiency and passage reading comprehension) separately, we repeated our analyses with each reading measure as the sole outcome in our models. The results showed that the effect of teachers' perceived ability on students' reading gains became statistically non‐significant when we used the two reading scores separately (see Tables S1 and S2). However, we note that this is likely due, at least in part, to the fact that measuring a construct with a single measure introduces more error than using a composite score derived from multiple measures (Cohen et al., 1996).

Finally, to explore whether the effect of teachers' perceived ability to teach reading on students' reading skills holds even when the effects of their school and the grade levels they teach were taken into account, we ran an additional set of multilevel models. The results showed that the effect of teachers' perceived ability became statistically non‐significant (β = .07, 95% CI [−.04, .18], *p* = .222) when the fixed effects of school and grade level were included in the models (see Table S3). We note, however, that this analysis might have inflated the risk of Type‐II error, given the relatively small sample size at the teacher level (*N* = 54).

Taken together, these results suggest that teachers' perceived ability to teach reading may be weakly associated with students' reading gains over time for the whole class, after controlling for other teacher variables and students' age, gender and earlier reading skills. In contrast, teachers' knowledge of dyslexia and warmth during teaching were not significantly associated with students' reading gains, either for the whole class or for lower‐performing children. However, these results should be viewed with caution, given our additional analyses showing that the effect of teachers' perceived ability on students' reading skills did not survive when examined separately for students with different performance levels or when controlling for the effects of school and grade level.

## DISCUSSION

In this study, we examined the roles of Chinese teachers' knowledge of dyslexia, perceived ability to teach reading and warmth during teaching in predicting their students' reading development. Our results showed that teachers' perceived ability to teach reading was a modest but significant predictor of their students' reading gains, even after controlling for their years of teaching experience, knowledge of dyslexia and warmth during teaching, as well as students' age, gender and earlier reading skills. This is consistent with findings from previous studies in Western contexts (e.g., Caprara et al., 2006; Parrila et al., 2024), providing evidence that the positive effects of teachers' teaching self‐efficacy can be observed in the Chinese context. Teachers who strongly believe in their expertise and ability to teach reading may be more likely to differentiate their instruction, monitor their students' progress and respond flexibly to their individual learning needs, all of which are crucial for fostering students' reading development (Guo et al., 2012; Tschannen‐Moran & Hoy, 2001).

On the other hand, teachers' knowledge of dyslexia was not significantly associated with their students' reading gains in our sample, either at the whole‐class level or among lower‐performing students. Although this finding contradicts our hypothesis and previous studies that have reported positive effects of teachers' knowledge of various language and literacy concepts (e.g., McCutchen et al., 2009; Parrila et al., 2024; Podhajski et al., 2009), we consider this null finding, particularly that for lower‐performing students, theoretically important because it indicates that dyslexia‐specific knowledge alone may not be sufficient for teachers to provide necessary support for students with reading difficulties. Our findings suggest that dyslexia‐specific knowledge may need to be integrated with broader knowledge of language and literacy concepts (e.g., phonology, morphology) in order to be effectively translated into classroom practice (Hudson et al., 2021; Parrila et al., 2024). Related to this, another possible reason for the null findings is that teachers' knowledge of dyslexia may not automatically inform their pedagogical decisions unless supported by appropriate professional development and instructional resources (Darling‐Hammond, 2012). Indeed, previous research has suggested that although teachers are often aware of students' reading difficulties, they may lack the procedural knowledge necessary to implement differentiated instructional strategies effectively (Moats, 2009; Washburn et al., 2011). Our findings suggest that similar challenges may exist in the Chinese educational context, where, unfortunately, opportunities for specialized pre‐ and in‐service teacher training to support students with dyslexia remain significantly limited.

In contrast to previous findings (e.g., Gregory & Korth, 2016; Pakarinen et al., 2017), teachers' warmth during teaching did not predict students' reading gains after accounting for teachers' years of experience, knowledge of dyslexia and perceived teaching ability. There are at least two explanations for these contrasting results. First, teachers' warmth during teaching may be associated with students' reading through engagement, attitude and motivation, rather than directly influencing their reading skills (Bergin & Bergin, 2009; Fall & Roberts, 2012; Ryan & Deci, 2000). Supporting this view, Hu et al. (2018) found that the effect of Chinese teachers' emotional support on their students' vocabulary was fully mediated by students' attitudes towards reading. Given the relatively short follow‐up period in the present study and our focus on specific reading outcomes, we may have been limited in our ability to detect these potential indirect effects. Future research would benefit from explicitly testing mediation models and from examining longer developmental intervals.

A second possible explanation relates to the pedagogical and structural background in which this study was conducted. In Chinese classrooms, teaching is usually highly structured and teacher‐centred, with instruction often delivered through whole‐class formats and limited opportunities for teacher–student interactions (Zhu & Li, 2019). This instructional framework, which prioritizes academic rigour and classroom order, may limit the frequency of the individualized teacher–student interactions through which warmth typically operates (Li, 2003; Tweed & Lehman, 2002). Consequently, even when teachers display warmth, students may be less likely to initiate interaction, ask questions or actively seek support (Guo et al., 2025). This may, in turn, constrain the potential impact of teachers' warmth on their students' learning.

Our results have several important educational implications. First, enhancing teachers' self‐efficacy (their belief in and confidence in their expertise and teaching ability) may be important for supporting primary school students' reading development in China. Educational authorities and policymakers may promote their teachers' self‐efficacy by providing professional development programmes that offer hands‐on practice, constructive feedback and support to strengthen their literacy instructional skills (e.g., Georgiou et al., 2020; Klassen & Chiu, 2010; Tschannen‐Moran & McMaster, 2009). Second, it seems unlikely that promoting teachers' knowledge of dyslexia alone will improve their students' reading outcomes, either at the whole‐class level or for lower‐performing students. Teachers need to be provided with a more comprehensive knowledge of broader language and literacy concepts, such as phonology and morphology; it would be equally important to provide them with more structured opportunities to apply this knowledge in daily instruction, such as using differentiated teaching strategies and targeted interventions for students who struggle with learning to read (Aaron et al., 2008; Parrila et al., 2024; Washburn et al., 2014).

Some limitations of our study are worth noting. First, we assessed teachers' knowledge of dyslexia using only four items, most of which focused on misconceptions about dyslexia. This limited scope may not have been sufficient to capture the breadth and depth of their understanding necessary to effectively support students' reading development. In addition, our teacher knowledge scale showed relatively low internal consistency (α = .62). Although the small number of items partly explains this value, the relatively low reliability may also suggest that measurement error attenuated associations and contributed to the null findings regarding its effect on students' reading. Future research should incorporate a more comprehensive set of items to provide a more robust assessment of teachers' knowledge of dyslexia. Second, we did not assess other important factors, such as teachers' quality of instruction, differentiation of instruction or students' reading attitudes, motivation and engagement, all of which may mediate the associations between teacher characteristics and students' reading outcomes (e.g., Jensen et al., 2019; Parrila et al., 2024). Third, because we used self‐report questionnaires to measure teachers' perceived ability to teach reading and warmth during teaching, we cannot rule out the possibility that these ratings overestimated their actual practices due to social desirability bias. This concern is particularly important given that a ceiling effect was observed in warmth during teaching, which may have restricted its variance and limited our ability to detect its associations with students' reading outcomes. Future studies could benefit from incorporating observational measures or student‐rated assessments to provide a more objective evaluation of teachers' practices.

To conclude, our findings add to a growing body of research examining the effect of various teacher characteristics on students' reading outcomes across languages and cultures. Our findings show that Chinese teachers' self‐efficacy (operationalized with perceived teaching ability), rather than knowledge of dyslexia and warmth during teaching, may play a role in promoting their students' reading gains. Beyond addressing a contextual gap, the present findings contribute to the broader literature by examining whether established theoretical links between teacher knowledge, perceived ability and relational warmth and students' reading outcomes generalize to a morphosyllabic writing system and a Confucian heritage educational context. Because reading acquisition and the manifestation of reading difficulties differ across orthographies, evidence from Chinese classrooms helps clarify the boundary conditions of models developed in Western contexts with alphabetic orthographies such as English. In doing so, this study extends understanding of how teacher‐level characteristics relate to students' reading development across linguistic and cultural settings. Future research should examine not only which aspects of teacher knowledge and instructional practices matter most, but also how and under what conditions these teacher characteristics can be effectively translated into classroom practice across different linguistic and sociocultural contexts.

## AUTHOR CONTRIBUTIONS

**Anna Jia‐Jun Zhang:** Funding acquisition; writing – original draft; writing – review and editing; conceptualization; methodology; formal analysis; project administration. **Tomohiro Inoue:** Supervision; writing – review and editing; methodology; conceptualization.

## FUNDING INFORMATION

This research was supported by the Children Growth Support Special Fund (WPEF‐YCGS‐011/24 to Anna Jia‐Jun Zhang) from Wenzhou People's Education Foundation.

## CONFLICT OF INTEREST STATEMENT

The authors have no conflict of interest to disclose.

## ETHICS APPROVAL

Ethics approval for the analyses has been obtained from the Survey and Behavioural Research Ethics Committee of The Chinese University of Hong Kong (Reference No. SBRE‐24‐0008).

## Supporting information


**Table S1.** Multilevel modelling for students' sentence reading efficiency.
**Table S2**. Multilevel modelling for students' passage reading comprehension.
**Table S3**. Multilevel modelling for students' reading skills with school and grade effects controlled.

## Data Availability

The authors do not have permission to share data at the level of individual participants. Pre‐registration of the analysis and all analysis codes, including the final model syntax specification and final model results that support the findings of the study, are available at https://osf.io/vuxh4/.
