# Supplementary material for: Are teachers' knowledge of dyslexia, perceived teaching ability and warmth during teaching related to Chinese students' reading development?
Source: Br J Educ Psychol. 2026 Jun 23;96(4):1574–92. doi: 10.1111/bjep.70103 (PMC13752643; doi:10.1111/bjep.70103)
Supplement: Supplementary file 1 [file BJEP-96-1574-s001.docx]

**Table S1**

*Multilevel Modelling for Students’ Sentence Reading Efficiency*

| *Fixed Effects* | Stand. estimate | *SE* | BC bootstrapped 95% CI | *p* |
| --- | --- | --- | --- | --- |
| *Student Level* |  |  |  |  |
| Intercept | 0.06 | 0.07 | [-0.08, 0.20] | .438 |
| Age_T1 | 0.13 | 0.05 | [0.02, 0.23] | .017 |
| Gender_T1^a^ | -0.07 | 0.02 | [-0.10, -0.03] | < .001 |
| Reading skills_T1 | 0.58 | 0.02 | [0.54, 0.62] | < .001 |
| *Teacher Level* |  |  |  |  |
| Knowledge of dyslexia_T1 | -0.03 | 0.07 | [-0.17, 0.11] | .682 |
| Perceived ability to teach reading_T1 | 0.14 | 0.07 | [0.00, 0.28] | .057 |
| Warmth during teaching_T1 | -0.01 | 0.07 | [-0.15, 0.13] | .851 |
| Years of teaching experience_T1 | 0.09 | 0.08 | [-0.06, 0.26] | .220 |
| *Random Effects* | Variance | *SD* |  |  |
| Intercept | 0.25 | 0.50 |  |  |
| Residual | 0.37 | 0.61 |  |  |
| *Model Metrics* | Value |  |  |  |
| Marginal *R*^2^ | 0.45 |  |  |  |
| Conditional *R*^2^ | 0.67 |  |  |  |

*Note.* ^a^ Students’ gender was coded as 1 for male and 0 for female.

**Table S2**

*Multilevel Modelling for Students’ Passage Reading Comprehension*

| *Fixed Effects* | Stand. estimate | *SE* | BC bootstrapped 95% CI | *p* |
| --- | --- | --- | --- | --- |
| *Student Level* |  |  |  |  |
| Intercept | 0.00 | 0.04 | [-0.07, -0.07] | .996 |
| Age_T1 | 0.16 | 0.04 | [0.09, 0.23] | <.001 |
| Gender_T1^a^ | -0.01 | 0.01 | [-0.03, 0.01] | .456 |
| Reading skills_T1 | 0.64 | 0.02 | [0.60, 0.68] | < .001 |
| *Teacher Level* |  |  |  |  |
| Knowledge of dyslexia_T1 | 0.03 | 0.04 | [-0.04, 0.10] | .390 |
| Perceived ability to teach reading_T1 | 0.07 | 0.04 | [-0.01, 0.14] | .078 |
| Warmth during teaching_T1 | -0.05 | 0.04 | [-0.13, 0.02] | .152 |
| Years of teaching experience_T1 | 0.12 | 0.04 | [0.04, 0.20] | .004 |
| *Random Effects* | Variance | *SD* |  |  |
| Intercept | 0.05 | 0.22 |  |  |
| Residual | 0.19 | 0.43 |  |  |
| *Model Metrics* |  |  |  |  |
| Marginal *R*^2^ | 0.69 |  |  |  |
| Conditional *R*^2^ | 0.75 |  |  |  |

*Note.* ^a^ Students’ gender was coded as 1 for male and 0 for female.

**Table S3**

*Multilevel Modelling for Students’ Reading Skills with School and Grade Effects Controlled*

| *Fixed Effects* | Stand. estimate | *SE* | BC bootstrapped 95% CI | *p* |
| --- | --- | --- | --- | --- |
| *Student Level* |  |  |  |  |
| Intercept | 0.01 | 0.15 | [-0.28, 0.31] | .962 |
| Age_T1 | 0.03 | 0.06 | [-0.08, 0.14] | .553 |
| Gender_T1^a^ | -0.04 | 0.01 | [-0.06, -0.01] | < .001 |
| Reading skills_T1 | 0.75 | 0.02 | [0.71, 0.79] | < .001 |
| *Teacher Level* |  |  |  |  |
| Knowledge of dyslexia_T1 | 0.01 | 0.05 | [-0.09, 0.10] | .854 |
| Perceived ability to teach reading_T1 | 0.07 | 0.06 | [-0.04, 0.18] | .222 |
| Warmth during teaching_T1 | -0.03 | 0.05 | [-0.13, 0.08] | .628 |
| Years of teaching experience_T1 | 0.13 | 0.06 | [0.02, 0.25] | .020 |
| *Random Effects* | Variance | *SD* |  |  |
| Intercept | 0.08 | 0.29 |  |  |
| Residual | 0.16 | 0.40 |  |  |
| *Model Metrics* | Value |  |  |  |
| Marginal *R*^2^ | 0.69 |  |  |  |
| Conditional *R*^2^ | 0.80 |  |  |  |

*Note.* ^a^ Students’ gender was coded as 1 for male and 0 for female.
